# Supplementary material for: Pharmacological prevention of bone loss and fractures following solid organ transplantations: Protocol for a systematic review and network meta-analysis
Source: PLoS One. 2024 Apr 26;19(4):e0302566. doi: 10.1371/journal.pone.0302566 (PMC11051654; doi:10.1371/journal.pone.0302566)
Supplement: S2 Table — (DOCX) [file pone.0302566.s002.docx]

**Table S2:** Ovid MEDLINE search strategy

| **Ovid MEDLINE® ALL, 1946 to February 01, 2024** | | |
| --- | --- | --- |
| **Line #** | **Search statement** | **Results** |
| 1 | Osteoporosis/ or Bone Resorption/ or Bone Diseases, Metabolic/ | 79438 |
| 2 | (osteoporos?s or osteop?enia).ti,ab. | 74482 |
| 3 | ((bone or osteoporotic) adj3 (loss or disease? or resorption or densit* or fragil* or demineral* or mineral* or decalcif* or calcif* or strength?)).ti,ab. | 147533 |
| 4 | "BMD".ti,ab. or Bone Density/ | 67823 |
| 5 | fracture?.ti,ab. or exp Fractures, Bone/ | 306968 |
| 6 | or/1-5 | 469574 |
| 7 | organ transplantation/ or heart transplantation/ or kidney transplantation/ or liver transplantation/ or lung transplantation/ or pancreas transplantation/ or heart-lung transplantation/ | 237937 |
| 8 | transplants/ or allografts/ or isografts/ or composite tissue allografts/ | 18074 |
| 9 | Transplant Recipients/ or transplant*.ti,ab. | 490685 |
| 10 | or/7-9 | 539110 |
| 11 | Bone Density Conservation Agents/ | 16284 |
| 12 | exp Diphosphonates/ or (bisphosphonate? or diphosphonate? or alendron* or clodron* or etidron* or Ibandron* or pamidron* or risedron* or medron* or zoledron*).ti,ab. | 36358 |
| 13 | Calcitonin/ or calcitonin.ti,ab. | 32492 |
| 14 | exp Vitamin D/ or (ergocalciferol or cholecalciferol or (vitamin D adj3 supplement*)).ti,ab. | 71973 |
| 15 | Calcium/ or (calcium adj3 supplement*).ti,ab. | 287449 |
| 16 | Raloxifene Hydrochloride/ or raloxifene.ti,ab. | 3932 |
| 17 | Teriparatide/ or (teriparatide or abaloparatide).ti,ab. | 2941 |
| 18 | Denosumab/ or (denosumab or romosozumab).ti,ab. | 3687 |
| 19 | or/11-18 | 409658 |
| 20 | randomized controlled trial.pt. | 606925 |
| 21 | controlled clinical trial.pt. | 95516 |
| 22 | random*.mp. | 1494133 |
| 23 | placebo.ti,ab. | 228483 |
| 24 | drug therapy.fs. | 2659853 |
| 25 | trial.ti,ab. | 683086 |
| 26 | or/20-25 | 4058997 |
| 27 | (6 or 19) and 10 and 26 | 1968 |
| 28 | exp animals/ not humans.sh. | 5189690 |
| 29 | 27 not 28 | 1745 |
