# Supplementary material for: Pharmacological prevention of bone loss and fractures following solid organ transplantations: Protocol for a systematic review and network meta-analysis
Source: PLoS One. 2024 Apr 26;19(4):e0302566. doi: 10.1371/journal.pone.0302566 (PMC11051654; doi:10.1371/journal.pone.0302566)
Supplement: Attachment — Submitted filename: Response to Reviewers.docx [file pone.0302566.s003.docx]

PLOS-ONE

1265 Battery Street, Suite 200

San Francisco, CA 94111

United States

March 5th, 2024

**Manuscript Number: PONE-D-24-04602**

**Manuscript Title:**

**Pharmacological prevention of bone loss and fractures following solid organ transplantations: Protocol for a systematic review and network meta-analysis**

**Dear reviewers**,

We would like to thank you for your time and insightful comments. We have attached our point-by-point to your comments below, as well as highlighting where the changes have been made in the manuscript to accommodate your suggestions.

**Response to Reviewer Comments:**

**Reviewer #1:**

1. **How do you plan to address potential confounding factors in your study, such as differences in patient demographics, comorbidities, and medication histories across the included trials?**

Thank you for your comment. To address potential confounding factors such as differences in patient demographics, comorbidities, and medication histories across the included trials, our revised protocol incorporates several strategies. We plan to implement subgroup analyses and meta-regressions if we detect significant inconsistencies in the analysis to better understand the influence of confounders on treatment outcomes. We have prospectively identified age, sex, and BMI as the three most important factors to assess based on review of prior literature. In addition, we will integrate one-stage sensitivity analysis in conjunction with E-value analog assessments to assess the impact of studies with high risks of bias.

Please find relevant edits in **MATERIALS AND METHODS > Heterogeneity and inconsistency assessment**:

“If inconsistency is identified, we will conduct network meta-regression models and subgroup analysis to identify how potential effect modifiers may impact our results. Based on previous literature reviews, our assessments for sources of inconsistency will be focused on age, sex, and BMI [21]. In addition, we will conduct one-stage sensitivity analysis with E-value analogs (if applicable) to evaluate the strength of unmeasured confounding [22] if we identified RCTs with a high risk of bias.”

2. **Could you elaborate on the rationale behind your choice of outcome measures, particularly in terms of their clinical relevance and sensitivity to detecting differences between treatment groups?**

Thank you for your comment. Fracture incidence was chosen as it is a patient-important outcome in the context of post-SOT bone loss and osteoporosis, as bone fractures have obvious functional and quality of life impacts on patients. BMD was chosen as a widely recognized and commonly reported surrogate measure of fracture risk, osteoporosis severity, and bone health. Patients with lower BMD are at higher risks of experiencing fractures. Both outcomes are commonly reported in osteoporosis research, and both outcomes have been shown to be differentially affected by different antiosteoporosis medications.

We have added passages to justify the choice of these outcomes in **MATERIALS AND METHODS > Outcome measures**:

“Fracture was chosen as a patient-important efficacy outcome for bone loss and osteoporosis, while BMD is chosen as a widely-recognized and commonly reported surrogate measure of fracture risk, osteoporosis severity, and bone health [13–15].”

3. **How will your study account for potential changes in clinical practice and the availability of new evidence during the analysis phase?**

We will proactively monitor literature sources for new publications throughout the review process and incorporate newly published data or conduct updated database searches, if needed. This is now being discussed in **MATERIALS AND METHODS > Study identification**:

“To account for potential changes in clinical practice and ensure that our findings are current, we will proactively monitor literature sources for new publications throughout the review process, and incorporate newly published data or conduct updated database searches, if needed.”

4. **In your discussion, you mentioned the importance of incorporating Chinese RCTs into your analysis. Could you discuss any challenges you anticipate in accessing and interpreting data from these studies, particularly in terms of language barriers and cultural differences in clinical practice?**

Our multinational research team includes members fluent in Chinese and comfortable navigating cultural nuances and clinical practices. As such, we do not anticipate any challenges in accessing and interpreting data from Chinese RCTs, with respect to language barriers and cultural differences in clinical practice. Immunosuppression and care guidelines post-SOT are also fairly standardized internationally, which may help mitigate differences in clinical practice between different countries. This is now discussed in the **DISCUSSION > Limitations**:

“Secondly, there may be certain differences in clinical practices adopted by RCTs originating from different countries that may impact our findings and contribute to higher heterogeneity. However, we expect to account for this by seeking expertise from our multinational and multilingual research team. This effect may also be mitigated by the existence of standardized international clinical practice guidelines for post-SOT care, which reduces the diversity in clinical practices among different geographical regions [39].”

5. **Could you discuss the potential limitations of your study?**

Thank you for noting this. We have revised our study protocol to acknowledge several potential limitations. Please see **DISCUSSIONS > Limitations**.

6. **The study protocol does not explicitly mention where all data underlying the findings will be made available when the study is complete. To ensure transparency and facilitate reproducibility, it would be advisable for the authors to explicitly state their plans for data sharing and accessibility in the final publication or in subsequent updates to the study protocol.**

We recognize the importance of data accessibility and reproducibility of research. Supplementary documents detailing our search strategy, data extraction forms, and statistical analysis will be made publicly available upon study completion. In addition, original data will be available upon request. This statement has been added in **MATERIALS AND METHODS > Data items**. We wish for other researchers to be able to analyze, replicate, and build upon our work.

**Reviewer #2: Minor suggestions:**

1. **It is preferable to mention which are your primary outcome(s) and which ones are your secondary outcomes.**

Thank you for highlighting this. To clarify, our primary outcome measure is the incidence of vertebral and nonvertebral fractures. Our secondary outcome measures will include: 1) changes in femoral neck, lumbar spine, and total hip BMD from baseline, 2) incidence of adverse events, and 3) incidence of serious adverse events (SAEs). We have revised the protocol to reflect this clarification; please see **MATERIAL AND METHODS > Outcome measures**.

2. **There is no mention of how articles published in languages other than English will be handled. This is particularly relevant for your study since you are searching a Chinese database.**

Please see Response to Reviewer #1, Comment 4.

3. **I suggest the addition of network graphs to visualize the overall structure of the comparisons in your network.**

Thank you for this suggestion. We planned to do this and have now added it to the protocol manuscript. Please see **MATERIALS AND METHODS > Network meta-analysis**:

“Network graphs will be provided to visualize the overall network structure of comparisons.”

4. **I am not familiar with this field of research, but I am wondering if treatment retention would be important information to extract and report.**

Thank you for this suggestion. We recognize the potential importance that treatment adherence and retention may play, and have amended the data extraction process to include this. We plan to report this in the final manuscript. Please see **MATERIALS AND METHODS > Data items**.
